# Mutational spectrum of tobacco associated oral squamous carcinoma and its therapeutic significance

**DOI:** 10.1186/s12957-019-1741-2

**Published:** 2019-11-27

**Authors:** Nishant Batta, Manoj Pandey

**Affiliations:** Department of Surgical Oncology, Institute of Medical Sciences, Banaras Hindu University, Varanasi, 221005 India

## Abstract

**Abstract:**

Oral squamous cell cancer (OSCC) is a common malignancy attributed to use of chewing smokeless tobacco and smoking. Most of the targeted strategies are based on EGFR expression and mutation; however, none of them has shown significant improvement in survival and response rates. We carried out this study to evaluate mutational profile of tobacco associated oral carcinoma with special emphasis on EGFR and its downstream events.

**Patients and methods:**

A total of 46 histologically proven cases were recruited between January 2017 and January 2019. Apart from detailed clinical and histological studies, the paraffin-embedded tissue was submitted for expression of 50 genes using Next Generation Sequencing using Ion Ampliseq Cancer Hotspot Panel v2.

**Results:**

The mean age of patients was 47.8 ± 10.9 years. Majority had tumors on buccal mucosa (24) and tongue (13). Nineteen of these tumors were larger than 4 cm, and 5 had adjacent site involvement. Thirty one were node positive. TP53 mutations were commonest seen in 19 followed by CDKN2A in 11, HRAS in 8, PIK3CA in 3, SMARCB1 in 2, and KIT, EGFR, BRAF, STK11, ABL1, RB1 in one case each. Concomitant TP53 mutation was identified with other mutations like CDKN2A, HRAS, KIT, PIK3CA, STK11, SMARCB1, ABL1, and RB1 making tobacco-associated OSCC as a heterogeneous mutational tumor with multiple events. A patient with TP53 mutations has poor disease free survival (47.4 vs 63% *p* = 0.17); however, this was not statistically significant.

**Conclusion:**

The study shows a heterogeneous mutational spectrum with multiple mutational events in OSCC. The low EGFR mutation rates and higher mutations in EGFR downstream pathways including that in TP53 and HRAS suggest that anti EGFR strategies may not succeed in these tumors and newer agents and therapeutic combinations need to be tried.

## Introduction

Oral squamous cell cancer (OSCC) is the most common malignancy among males in India and the eighth most common cancer worldwide [1]. In India, gingivobuccal region of oral cavity is predominantly affected, comprising buccal mucosa and lower gums, whereas in the west, tongue is the most commonly involved subsite of oral cancer [2, 3]. The risk factors for OSCC involve an interaction between the habits, environmental (tobacco, betel quid, alcohol, HPV, etc.), and genetic (EGFR, TP53, CDKN2A, etc.) factors [4–8].

EGFR is a member of receptor protein tyrosine kinase family with 42–80% over expression in head neck squamous cell carcinoma (HNSCC), whereas EGFR gene amplification is seen in up to 30% of HNSCC, and yet the results of EGFR targeting are not satisfactory. Since the approval of EGFR targeting drug, cetuximab for locally or regionally advanced and for metastatic HNSCC a lot of data has been generated on its use [9–12]. The EXTREME trial showed some treatment success with cetuximab plus platinum based chemotherapy in HNSCC; however, EGFR expression level was not found to be clinically useful predictive biomarker [13]. In platinum-refractory HNSCC, the response rate with cetuximab monotherapy is only 10% [14]. SPECTRUM trial compared cisplatin/5-FU plus panitumumab to cisplatin/5-FU alone in patients with metastatic/recurrent SCCHN and showed significant activity of panitumumab [15]. Despite near universal expression of EGFR in HNSCC, there is only modest activity of these monoclonal antibodies.

Other than anti-EGFR monoclonal antibodies (mAb), EGFR tyrosine kinase inhibitors (TKI) have also been tried with mixed success. Afatinib (selective EGFR and HER2 inhibitor), erlotinib (an oral reversible EGFR TKI), and gefitinib (a reversible EGFR TKI) have been used in cases with EGFR exon 19 deletions or exon 21 (L858R) substitution mutations as detected by an FDA-approved test. Vamurafenib (an oral selective inhibitor of BRAF kinase V600E oncogene) in unresectable and metastatic melanoma with the BRAFV600E mutation have been recently approved [11–16]. But still mono therapy with TKI’s has only modest activity in EGFR mutated HNSCC [17].

Specific genetic mutations in HNSCC had been identified by next generation sequencing (NGS), some of which are potential targets and therapies can be tailored to augment existed EGFR targeted therapies [18]. The earlier results have shown mutation of the TP53, CDKN2A, HRAS, and PIK3CA genes [19–21] in downstream EGFR pathways, and hence can explain moderate activity of anti EGFR therapies in OSSC.

The Cancer Genome Atlas (TCGA) data profiling 279 HNSCCs showing mutations of the oncogene PIK3CA, novel alterations involving loss of TRAF3, and amplification of the cell-cycle gene E2F1 [22]. It also showed that tobacco-associated tumors showed high frequency of mutations in TP53 and CDKN2A [22]. Further a mutation-based signature affecting ten genes (HRAS, BRAF, FGFR3, SMAD4, KIT, PTEN, NOTCH1, AKT1, CTNNB1, and PTPN11) had been found to predict disease free survival (DFS) [19]. Despite identification of over 1500 mutation signatures in various genes in HNSCC, the data is still insufficient to plan therapeutic strategies based on these, and there is need to identify and establish potential genetic biomarkers and targets.

Further, personalized medicine, identification of optimal responders to targeted therapy, and creation of large databases integrating clinical and genetic data will help provide a panel of useful predictive biomarkers that will subsequently change clinical practices, and this study is a small step in this direction.

## Patients and methods

This study was conducted in the Department of Surgical Oncology, Institute of Medical Sciences, Banaras Hindu University. Histologically proven cases of primary OSCC with history of tobacco intake were included, and patients with history of prior chemotherapy (for any reason) or radiotherapy were excluded. After the approval of the ethical committee and obtaining the consent, patients were recruited between 1st January 2017 and 1st January 2019.

Comprehensive history and physical examination was taken, and all the details were recorded in the preset pro forma. A biopsy to establish the diagnosis and CT scan of the head and neck to measure the tumor dimensions and stage the disease were performed before initiation of treatment. After the surgery the specimen was submitted for detailed histopathological examination. The archival tissue (formalin-fixed paraffin-embedded) was studied for expression of 50 genes by molecular analysis using next generation sequencing.

### Next generation sequencing

#### DNA isolation/extraction

DNA isolation from FFPE tissue with deparaffinization using ReliaPrep™ FFPE gDNA Miniprep System, Promega Corporation, India, was carried out following the manufacturer’s instruction protocol. For sections ≤ 50 μm, 300 μl of mineral oil and for sections > 50 μm, 500 μl of mineral oil was used for deparaffinization and incubate at 80 °C for 1 min. Added 20 μl of proteinase K directly to the lower phase and mixed by pipetting and incubate at 56 °C for 1 h and then at 80 °C for 4 h. The sample was allowed to cool at room temperature. 10 μl of RNase was added to the lysed sample in the lower phase. The lower phase was mixed by pipetting and incubated at room temperature (20–25 °C) for 5 min.

#### Template preparation

Template preparation was done by building a library of nucleic acids (DNA or complementary DNA (cDNA) and amplifying that library. DNA Library preparation was carried out using an Ion PGM™ System, (Thermo Fisher Scientific, San Francisco, CA, USA) according to the manufacturer’s instructions. Bar coded libraries using Ion Ampliseq Cancer Hotspot Panel v2 (Thermo Fisher Scientific) research panel were created. The Ion AmpliSeq™ Cancer Hotspot Panel v2 was designed to amplify 207 amplicons covering approximately 2800 COSMIC mutations from 50 oncogenes and tumor suppressor genes (ABL1, EGFR, GNAS, KRAS, PTPN11, AKT1, ERBB2, GNAQ, MET, RB1, ALK, ERBB4, HNF1A, MLH1, RET, APC, EZH2, HRAS, MPL, SMAD4, ATM, FBXW7, IDH1, NOTCH1, SMARCB1, BRAF, FGFR1, JAK2, NPM1, SMO, CDH1, FGFR2, JAK3, NRAS, SRC, CDKN2A, FGFR3, IDH2, PDGFRA, STK11, CSF1R, FLT3, KDR, PIK3CA, TP53, CTNNB1, GNA11, KIT, PTEN, VHL) with specific coverage of KRAS, HRAS, NRAS, BRAF, and EGFR exon 18–21with performance of at least 2000× sequence coverage for eight samples on one Ion 316 chip. In addition, the primers in this panel were designed to produce, on average, 154-bp amplicons, so that even degraded samples were used to generate reliable data. Bar coded libraries were combined to a final concentration of 100 pM. The next step was amplification of libraries which was done by using emulsion PCR (emPCR) on the 2 Ion OneTouch system (Life Technologies).

#### Sequencing and imaging

The Ion PGM^TM^ Torrent relied on the library fragments which acted as a template, off which a new DNA fragment was synthesized. As nucleotides were incorporated into the growing DNA strand, they were digitally recorded as sequence. Sequencing primer and polymerase were added to the final enriched ISPs prior to loading onto 316 (100 Mb output) chips. Alignment of sequences was performed using Torrent Suite™ Software (version5.2.0) on the Ion PGM™ Torrent Server.

#### Sequencing data analysis

After sequencing, preprocessing the data to remove adapter sequences and low-quality reads was carried out followed by mapping of the data to a reference genome or de novo alignment of the sequence reads, and analysis of the compiled sequence using wide variety of bioinformatics assessments, including genetic variant calling for detection of SNPs or indels (i.e., the insertion or deletion of bases), detection of novel genes or regulatory elements, and assessment of transcript expression levels. Data analysis was carried out with Torrent Suite Software V.5.2.0 (Life Technologies). The Ion Reporter suite (Life Technologies) was used to filter polymorphic variants.

### Statistical analysis

Statistical analysis was performed using SPSS version 23.0 (IBM Corp., Armonk, NY). The baseline characteristics were compared using the chi-square and Fisher Exact test. Survival was estimated using Kaplan-Meier method and was compared using log-rank test. Statistical significance was set at *P* < 0.05.

## Results

The mean age of patients was 47.8 ± 10.9 years. Majority of cases were male (93.5%) followed by females with male: female ratio of 14.3:1. Only 1 patient had family history of cancer. Leukoplakia was present in 2 (4.3%) cases, and others had no precancerous lesion. The clinical and histopathological findings and treatment is presented in Table 1.

**Table 1:** Clinical and histopathological findings

Clinical findings	Frequency	Percent
Site of tumor		
Tongue	13	28.3
Buccal Mucosa	24	52.2
Lower Alveolus	6	13.0
Upper Alveolus	1	2.20
Lip	2	4.30
Side		
Right	18	39.1
Left	26	56.5
Midline	1	2.20
Crossing midline	1	2.20
Number lesion		
Single	44	95.70
Multiple	2	4.30
Clinical size of tumor (cm)*		
≥ 4	19	41.30
< 4	27	58.70
Extension to adjacent site		
Yes	17	37
No	29	63
T status		
T2	23	50.00
T3	8	17.40
T4	15	32.60
Clinical nodes		
No	15	32.60
N1	17	37.00
N2	13	28.30
N3	1	2.20
Stage		
II	17	37.00
III	11	23.90
IVa	17	37.00
IVb	1	2.20
Comorbid factors		
Yes	8	17.40
No	38	82.60
ECOG performance status		
0	9	19.60
1	37	80.40
Imaging		
CT size (cm)		
< 4	13	28.30
≥ 4	33	71.70
Neck node present	22	47.80
Involved adjacent site		
Bone	9	19.60
Skin	5	10.90
Muscle	9	19.60
TNM stage (CT)		
II	11	23.90
III	17	37.00
IVa	17	37.00
IVb	1	2.00
Neck dissection		
SOHND	22	47.80
MRND	17	37.00
None	7	15.20
Surgical reconstruction		
None	16	34.80
Pectoralis major myocutaneous flap	14	30.40
Nasolabial flap	13	28.30
Buccal pad of fat	2	4.30
Sternocleidomastoid flap	1	2.20
Total	46	100.0
Histopathological findings		
HPE grade		
I (well differentiated)	22	47.80
II (moderately differentiated)	22	47.80
III (poorly differentiated)	2	4.30
T size (cm)		
≥ 4	11	23.90
< 4	35	76.10
Positive margin	2	4.30
Lymphovascular invasion	13	28.30
Perineural invasion	20	43.50
Depth of invasion (mm)		
< 5	4	8.70
≥ 5 to < 10	24	52.20
≥ 10	18	39.10
Pathological stage		
I	1	2.20
II	22	47.80
III	7	15.20
IVa	13	28.30
IVb	3	6.50
Mutations		
TP53	19	41.3
CDKN2A	11	23.9
HRAS	8	17.4
PIK3CA	3	6.5
SMARCB1	2	4.3
KIT	1	2.2
EGFR	1	2.2
BRAF	1	2.2
STK11	1	2.2
ABL1	1	2.2
RB1	1	2.2
NRAS	0	0.0
KRAS	0	0.0

*The largest numeric value of length, breadth, and depth had been considered

Out of 46 cases, 19 (41.3%) had received adjuvant radiotherapy, and 8 (17.4%) had received adjuvant chemotherapy. There were total 20 recurrences, 17 recurrences occurred at primary site, 2 were the lymph node recurrences, and 1 was the second primary. All stage III, IVa, and IVb were advised adjuvant radiotherapy, but 6 cases did not take, of these 2 cases had recurrence at primary site which could not be re-excised and were started on palliative chemotherapy. Out of 19 cases who were given adjuvant radiotherapy in 9 had loco-regional recurrence.

TP53 was the most common (41.3%) mutation followed by CDKN2A (23.9%), HRAS (17.4%), PIK3CA (6.5%), SMARCB1 (4.3%), and KIT, EGFR, BRAF, STK11, ABL1, RB1 (2.2% each). The relationship of TP53, CDKN2A, PIK3CA, and HRAS mutation with clinical-pathological factors are presented in Table 2. The HRAS and PIK3CA mutation had significant association with site of tumor, i.e., lower lip (*p* = 0.002) and lower alveolus (*p* = 0.004).

**Table 2:** Relationship of TP53, CDKN2A, PIK3CA, HRAS mutation with clinical-pathological factors

	TP53		*p* value	CDKN2A		*p* value	PIK3CA		*p* value	HRAS		*p* value
	Positive (*n* = 19)	Negative (*n* = 27)		Positive (*n* = 11)	Negative (*n* = 35)		Positive (*n* = 3)	Negative (*n* = 43)		Positive (*n* = 8)	Negative (*n* = 38)	
HPE size (cm)												
≥ 4 (*n* = 10)	8	3	0.032*	3	8	0.526	2	9	0.073	1	10	0.405
< 4 (*n* = 26)	11	24		8	27		1	34		7	28	
Site												
Tongue			0.462			0.330			0.261			0.882
Yes	6	7		2	11		0	13		2	11	
No	13	20		9	24		3	30		6	27	
Buccal mucosa			0.402			0.301			0.499			0.892
Yes	9	15		7	17		1	23		4	20	
No	10	12		11	18		2	20		4	18	
Lower alveolus			0.484			0.555			0.004*			0.228
Yes	3	3		1	5		2	4		0	6	
No	16	24		10	30		1	39		8	32	
Upper alveolus			0.413			0.239			1.000			1.000
Yes	1	0		1	0		0	1		0	1	
No	18	27		10	35		3	42		8	37	
Lower lip			0.339			1.000			1.000			0.002*
Yes	0	2		0	2		0	2		2	0	
No	19	25		11	33		3	41		6	38	
HPE grade												
Moderately differentiated	10	10	0.337	3	17	0.371	2	18	0.686	3	17	0.705
Well differentiated	9	15		7	17		1	23		5	19	
Poorly differentiated	0	2		1	1		0	2		0	2	
Vascular invasion												
Yes	7	6	0.225	2	11	0.330	1	12	1.000	2	11	0.882
No	12	21		9	24		2	31		6	27	
Perineural invasion												
Yes	9	11	0.442	3	17	0.187	3	17	0.041*	2	18	0.246
No	10	16		8	18		0	26		6	20	
Depth invasion												
< 5	3	1	0.143	1	3	0.642	0	4	0.571	0	4	0.316
5–10	7	17		7	17		1	23		6	18	
> 10	9	9		3	15		2	16		2	16	
Lymph node positivity												
Yes	6	8	0.570	3	11	0.556	1	13	0.910	3	11	0.633
No	13	19		8	24		2	30		5	27	
Extra nodal extension												
Yes	2	5	0.457	1	6	0.459	0	7	1.000	1	6	1.000
No	17	22		10	29		3	36		7	32	
T status												
T2	8	15	0.660	8	15	0.127	0	23	0.036*	5	18	0.057
T3	4	4		0	8		0	8		3	5	
T4	7	8		3	12		3	12		0	15	
Stage												
I	1	0	0.453	0	1	0.282	0	1	0.086	0	1	0.880
II	9	13		8	14		0	22		4	18	
III	3	4		0	7		0	7		1	6	
IVa	6	7		2	11		3	10		3	10	
IVb	0	3		1	2		0	3		0	3	

*statistically significant

**Fig. 1:**
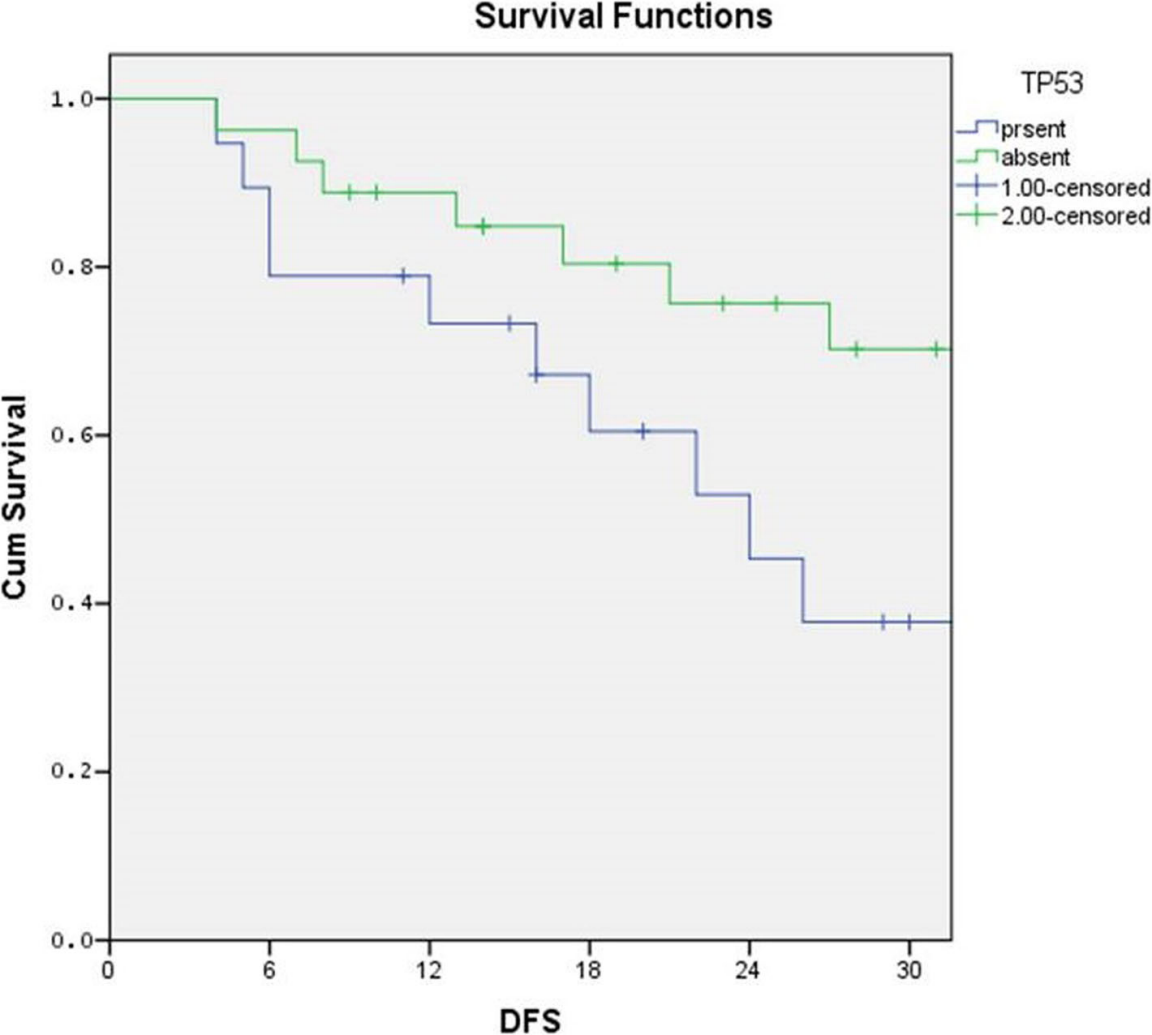
Kaplan-Meier survival curve showing difference in survival between p53 mutated and wild groups. The 18 months DFS rates were 53.3% and 71.4% for TP53 mutation present and absent respectively (log rank = 1.44; *p* = 0.229)

The median follow-up in this study is 19.3 months, and the median disease free survival was 16 months. The 30 months OS rates and DFS rates of TP53, CDKN2A, PIK3CA, and HRAS is depicted in Table 3, the details of the identified mutation are recorded in Table 4. The disease free survival rates were 47.4% and 63% for TP53 mutation present and absent respectively (Log rank = 1.44; *p* = 0.17) (Fig. 1).

**Table 3:** Disease free survival and overall survival of various clinicopathological factors and studied mutations

Variables	30 month disease free survival (%)	*p* value	30 month overall survival (%)	*p* value
Age (year)		0.001*		0.003*
< 46	36.4		45.5	
> 46	75.0		87.5	
Site of tumor		0.641		0.665
Tongue	61.5		69.2	
Buccal mucosa	50.0		66.7	
Lower alveolus	66.7		50.0	
Upper alveolus	100		100	
Lower lip	50.0		100	
HPE size (cm)		0.846		0.720
< 4	54.3		74.3	
≥ 4	63.6		45.5	
Lymph node (HPE)		0.250		0.021*
Positive	42.9		42.9	
Negative	62.5		78.1	
Extra nodal extension (ENE)		0.464		0.997
Present	42.9		71.4	
Absent	59.0		66.7	
Depth of invasion (mm)		0.731		0.008*
< 5	50.0		100	
≥ 5, > 10	58.3		79.2	
< 10	55.6		44.4	
Vascular invasion		0.018*		0.042*
Positive	23.1		38.5	
Negative	69.7		78.8	
PNI		0.196		0.134
Positive	40.0		50.0	
Negative	69.2		80.8	
Adjuvant RT		0.731		0.042*
Yes	52.6		47.4	
No	59.3		81.5	
Mutations				
TP53		0.176		0.082
Present	47.4		52.6	
Absent	63.0		77.8	
CDKN2A		0.320		0.431
Present	72.7		81.8	
Absent	51.4		62.9	
PIK3CA		0.148		0.221
Present	100		100	
Absent	53.5		65.1	
HRAS		0.629		0.761
Present	62.5		75.0	
Absent	55.3		65.8	

*statistically significant

**Table 4:** Mutational analysis of all study subjects

S. No.	Mutations	Cytogenetic location	Codon no.	Variant	Variant type	Site
1	TP53	17p13.1	Codon 72	c.215C>G (Pro72Arg)	Mis-sense Transversion	Buccal mucosa
2	TP53	17p13.1	Codon 72	c.215C>G (Pro72Arg)	Mis-sense Transversion	Buccal mucosa
3	TP53	17p13.1	Codon 72	c.215C>G (Pro72Arg)	Mis-sense Transversion	Tongue
4	TP53	17p13.1	Codon 72	c.215C>G (Pro72Arg)	Mis-sense Transversion	Tongue
5	TP53	17p13.1	Codon 72	c.215C>G (Pro72Arg)	Mis-sense Transversion	Buccal mucosa
6	TP53	17p13.1	Codon 152	c.455C>T (Pro152Leu)	Mis-sense Transition	Lower alveolus
7	TP53	17p13.1	Codon 158	c.472C>T (Arg158Cys)	Mis-sense Transition	Tongue
8	TP53	17p13.1	Codon 165	c.493C>T (Gln165Ter)	Non-sense	Tongue
9	TP53	17p13.1	Codon 173	c.517G>A (Val173Met)	Mis-sense Transition	Tongue
10	TP53	17p13.1	Codon 175	c.524G>A (Arg175His)	Mis-sense Transition	Buccal mucosa
11	TP53	17p13.1	Codon 204	c.610G>T (Glu204Ter)	Non-sense	Buccal mucosa
12	TP53	17p13.1	Codon 213	c.638G>A (Arg213Gln)	Mis-sense Transition	Buccal mucosa
13	TP53	17p13.1	Codon 241	c.722C>T (Ser241Phe)	Mis-sense Transition	Lower alveolus
14	TP53	17p13.1	Codon 244	c.731G>C (Gly244Ala)	Mis-sense Transversion	Buccal mucosa
15	TP53	17p13.1	Codon 248	c.742C>T (Arg248Trp)	Mis-sense Transition	Buccal mucosa
16	TP53	17p13.1	Codon 248	c.742C>T (Arg248Trp)	Mis-sense Transition	Tongue
17	TP53	17p13.1	Codon 248	c.743G>A (Arg248Gln)	Mis-sense Transition	Buccal mucosa
18	TP53	17p13.1	Codon 267	c.799C>T (Arg267Tryp)	Mis-sense Transition	Tongue
19	TP53	17p13.1	Codon 274	c.820G>T (Val274Phe)	Mis-sense Tranversion	Lower alveolus
20	TP53	17p13.1	Codon 274	c.821T>G (Val274Gly)	Mis-sense Transversion	Buccal mucosa
21	TP53	17p13.1	Codon 301	c.902delC (pro301Glnfs)	Deletion (frameshift)	Tongue
22	TP53	17p13.1	Codon 306	c.916C>T (Arg306Ter)	Non-sense	Buccal mucosa
23	TP53	17p13.1	Codon 306	c.916C>T (Arg306Ter)	Non-sense	Upper alveolus
24	TP53	17p13.1	-	c.376-1G>A unknown	Intron variant (splice site)	Buccal mucosa
25	CDKN2A	9p21.3	Codon78	c.233-234delTC (Leu78fs)	Deletion (frameshift)	Buccal mucosa
26	CDKN2A	9p21.3	Codon 80	c.238 C>T (Arg80Ter)	Non-sense	Buccal mucosa
27	CDKN2A	9p21.3	Codon 80	c.238 C>T (Arg80Ter)	Non-sense	Buccal mucosa
28	CDKN2A	9p21.3	Codon 80	c.238C>T (Arg80Ter)	Non-sense	Buccal mucosa
29	CDKN2A	9p21.3	Codon 80	c.238C>T (Arg80Ter)	Non-sense	Tongue
30	CDKN2A	9p21.3	Codon 80	c.238C>T (Arg80Ter)	Non-sense	Lower alveolus
31	CDKN2A	9p21.3	Codon 80	c.238C>T (Arg80Ter)	Non-sense	Tongue
32	CDKN2A	9p21.3	Codon 110	c.330G>A (Trp110Ter)	Non-sense	Buccal mucosa
33	CDKN2A	9p21.3	Codon 110	c.330G>A (Trp110Ter)	Non-sense	Buccal mucosa
34	CDKN2A	9p21.3	Codon 110	c.330G>A (Trp110Ter)	Non-sense	Upper alveolus
35	HRAS	11p15.5	Codon 12	c.34G>A (Gly12Ser)	Mis-sense Transition	Buccal mucosa
36	HRAS	11p15.5	Codon 12	c.34G>A (Gly12Ser)	Mis-sense Transition	Buccal mucosa
37	HRAS	11p15.5	Codon 12	c.34G>A (Gly12Ser)	Mis-sense Transition	Buccal mucosa
38	HRAS	11p15.5	Codon 12	c.35G>A (Gly12Asp)	Mis-sense Transition	Lower lip
39	HRAS	11p15.5	Codon 13	c.37G>C (Gly13Arg)	Mis-sense Transversion	Lower lip
40	HRAS	11p15.5	Codon 13	c.38G>T (Gly13Val)	Mis-sense Transversion	Buccal mucosa
41	HRAS	11p15.5	Codon 61	c.181C>A (Gln61Lys)	Mis-sense Transversion	Tongue
42	HRAS	11p15.5	Codon 61	c.182A>T (Gln61Leu)	Mis-sense Transversion	Tongue
43	PIK3CA	3q26.32	Codon 542	c.1625A>C (Glu542Ala)	Mis-sense Transversion	Lower alveolus
44	PIK3CA	3q26.3	Codon 542	c.1624G>A (Glu542Lys)	Mis-sense Transition	Lower alveolus
45	PIK3CA	3q26.3	Codon 1047	c.3140 A>G (His1047Arg)	Mis-sense Transition	Buccal mucosa
46	SMARCB1	22q11.23	-	c.1146-41G>A unknown	Intron variant	Buccal mucosa
47	SMARCB1	22q11.23	-	c.1146-41G>A	Intron variant	Buccal mucosa
48	KIT	4q12	Codon 541	c.1621A>C (Met541Leu)	Mis-sense Transversion	Buccal mucosa
49	BRAF	7q34	Codon 466	c.1397G>C (Gly466Ala)	Mis-sense Transversion	Lower alveolus
50	STK11	19p13.3	Codon 357	c.1071G>T (Glu357Asp)	Mis-sense Transversion	Lower alveolus
51	ABL1	9q34.12	Codon 274	c.764A>T (Glu274Val)	Mis-sense Transversion	Tongue
			Codon 415	c.1187A>G (His415Pro)	Mis-sense Transition	
52	EGFR	7p11.2	Codon 746	c.2234delAGG (Glu746del)	Deletion (in frame Deletion)	Tongue
53	RB1	13q14.2	Codon 680	c.2039T>C (Ile680Thr)	Mis-sense Transition	Tongue

## Discussion

Incidence of OSCC has increased with widespread use of smokeless tobacco, betel quid, HPV infection, environmental pollution, and genetic risk factors. Anatomically, the oral cavity is composed of the mucosal lip, oral tongue, floor of mouth, mandibular and maxillary gingiva, retromolar trigone, buccal mucosa, and hard palate subsites controlling the speech, swallowing, and facial projections. Surgery is the mainstay treatment for OSCC, despite current advances in the treatment options, the fatality of oral cancer has remained mostly unchanged. In addition to radiotherapy, anti-EGFR strategies have shown improved outcomes in the adjuvant setting and have become an active area of research.

The use of next generation sequencing (NGS) in HNSCC has led to identification of novel mutated tumor suppressor genes (TP53, CDKN2A) and oncogenes (PIK3CA, HRAS, EGFR) and has led to the development of predictive biomarkers. There are several genetic alterations responsible for development of OSCC; out of which, EGFR is a validated target, other than PI3K, PTEN, VEGF, JAK-STAT, etc. However, in presence of downstream mutations the EGFR targeting is mostly ineffective.

In our study, concomitant TP53 mutation was present with other mutations like CDKN2A, HRAS, KIT, PIK3CA, STK11, SMARCB1, ABL1, and RB1 making oral cavity squamous cell cancer as a heterogeneous mutational tumor. There were 16 different TP53 mutations in exons 4–8, out which most commonly was found on codon 72, c.215C>G (Pro72Arg) transversion variant in 5 patients (Table 4). This variant had been demonstrated in oral cavity squamous cell cancers (OSCC) previously [23]. Similarly codon 248 c.743G>A (Arg248Gln) and codon 306 c.916C>T (Arg306Ter) had been reported earlier [24]. An intron splice variant c.376-1G>A [25] and mutations in codon 152 [26], 158 [27], 165 [28], 204 [29], and 241 [30] were also previously reported in OSCC. Whereas codon 244 c.731G>C (Gly244Ala) and codon c.799C>T (Arg267Tryp) had never been reported in OSCC but has been reported in other cancers [31, 32]. Mutation on codon 274 c.821T>G (Val274Gly) and a deletion frameshift variant at codon 301 (Pro301Glnfs) had also never been reported in OSCC or in other cancers and were a novel finding in this study.

EGFR point mutations are reported in 9% patients in a study conducted by Dubot et al. in 2017 [33]; however, we have identified only one EGFR mutation on exon 19, codon 746 (Glu746del) which is an in-frame deletion mutation. This mutation has been previously reported by Ragga et al. in 2006 in HNSCC [34].

The point mutations that activate RAS protooncogenes have been found to be located mainly at the codons 12, 13, and 61 [35] and is estimated to be 15% [36]. In our study, the HRAS found to be mutated in 17.4% patients. All these mutations are previously reported in OSCC and have high incidence in lip vermilion cancers [37–39].

V600E mutation is the most frequently identified cancer-causing mutations in melanoma and other malignancies like non-Hodgkin lymphoma, colorectal cancer, thyroid carcinoma, non-small cell lung carcinoma, hairy cell leukemia, and adenocarcinoma of lung. In our study, we found a transversion mutation in BRAF exon11 codon 466 c.1397G>C (Gly466Ala), in a patient of carcinoma lower alveolus. This mutation has been reported in COSMIC database and CLINVAR database within exons 11 and 15 with a predominant nucleotide change at codons 599 and 468 in HNSCC (pharynx) [40]. Another study reported BRAF mutation in exon15, codon 412 in maxillary alveolus [41]. After having a thorough search of genetic mutation databases, we found that the mutation in BRAF exon 11 c.1397G>C (Gly466Ala) has never been reported in lower alveolus.

Of the 11, CDKN2A mutations identified in this study, 10 were non-sense mutations, and one was frameshift deletion. The most common mutation which was present in 6 patients, i.e., c.238C>T (Arg80Ter) was found to be frequently reported mutation in OSCC. Similarly, c.330G>A (Trp110Ter) present in three patients was also found to be a known mutation in oral cavity SCC [42]. Further, we found a novel mutation on codon 78, c.233-234delTC (Leu78fs) in a patient of carcinoma buccal mucosa.

The frequency of mutations of the PIK3CA gene has been reported in 11% HNSCC [43]. Kozaki et al. did mutational analysis of cell lines and primary tumors of OSCC, found a significant correlation between the advanced stage of OSCC and the frequency with which PIK3CA is mutated in exons 9 and 20 [44]. PIK3CA mutation was the most common mutation in HPV positive HNSCC while phosphate and tensin homolog (PTEN) loss were frequent event independent of HPV status [45, 46]. PIK3CA is reported to be mutated in 12% − 16% of HNSCC exome/genome analysis. In our study, percentage of PIK3CA mutation was 6.5% and was significantly associated with lower alveolus lesions. The codon variant His1047Arg and Glu542Lys have been reported in many studies as hotspot mutation sites in HNSCC, but Glu542Ala is only reported in cancers of the breast, endometrium, prostate and esophagus [47] and is for the first time being reported in OSCC.

KIT gene mutations have earlier been reported in gastrointestinal stromal tumors, chronic myeloid leukemia, etc .[48],but has never been reported in OSCC; however, it was found in one of our patients at exon 10, codon 541 c.1621A>C (Met541Leu) as transversion mutation.

STK11 is a tumor-suppressor gene involved in causing Peutz Jeghers syndrome, but the role of STK11/LKB1 gene inactivation in neoplasia has not been conclusively demonstrated so far. Tan et al. conducted a study in 2014 on carcinoma tongue patients, compared their genetic mutations with Lung Carta 1.0 gene panel and found 9% patients had STK11 mutations [49]. In our study, only one patient had STK11 mutation exon 8 codon 357 c.1071G>T (Glu357Asp) transversion variant type that is different from those reported by Tan et al. and has never been reported before in OSCC.

SMARCB1 mutation c.1146-41G>A as intron variants were present in two patients which were also having Tp53 mutation along with, these mutations are not known to play any major role in tumorigenesis. Two ABL1 mutations were present in one patient of carcinoma tongue along with RB and TP53 mutations. ABL1 c.764A>T (Glu274Val) and c.1187A>G (His415Pro) mutations are commonly seen in patients of chronic myeloid leukemia and have never been reported in oral cavity squamous cell cancers. RB mutation is only reported in cases of carcinoma breast and thyroid, but we found a novel mutation c.2039T>C (Ile680Thr) in one of our patients.

The results of the present study suggest that the mutation spectrum of OSCC may be different in different races, with Indian OSCC showing some distinct mutations that has not been seen in Chinese and Caucasians reported earlier. It also show that the mutations vary by subsite within the oral cavity, though TP53, CDKN2A, and PIK3CA mutations could be the common event in all oral cavity subsites [50–52]. Despite the limitations of the sample size this study shows that mutations in tobacco associated cancers are high, and concomitant multiple mutations are a common phenomenon. Low rate of EGFR mutations and higher mutations in EGFR downstream pathways like those in TP53, HRAS, etc., suggest that anti EGFR strategies may not be very effective against OSCC, and there is need to identify more suitable targets.

## Conclusion

The present study shows a higher incidence of mutations in tobacco-associated Indian OSCC, with presence of more than one mutation in most cases. Demonstration of downstream mutations in p53 and RAS provide evidences as to why the EGFR strategies are not effective in these patients, suggesting the role of combination of strategies or selection of strategies based on identifiable genetic mutations.

## Data Availability

Hard and soft copy of data is available with authors, and blinded data can be provided on reasonable requests within the provisions of Indian law.
